# The Molecular Assembly State of Drp1 Controls its Association With the Mitochondrial Recruitment Receptors Mff and MIEF1/2

**DOI:** 10.3389/fcell.2021.706687

**Published:** 2021-11-05

**Authors:** Rong Yu, Shao-Bo Jin, Maria Ankarcrona, Urban Lendahl, Monica Nistér, Jian Zhao

**Affiliations:** ^1^ Department of Oncology-Pathology, Karolinska Institutet, BioClinicum, Karolinska University Hospital Solna, Solna, Sweden; ^2^ Department of Cell and Molecular Biology, Karolinska Institutet, Biomedicum, Stockholm, Sweden; ^3^ Department of Neurobiology, Care Sciences and Society, Center for Alzheimer Research, Division of Neurogeriatrics, Karolinska Institutet, BioClinicum, Solna, Sweden

**Keywords:** Drp1, Drp1 mutation, Mff, MIEF1, MIEF2, oligomerization, mitochondria, mitochondrial dynamics

## Abstract

Drp1 is a central player in mitochondrial fission and is recruited to mitochondria by Mff and MIEFs (MIEF1 and MIEF2), but little is known about how its assembly state affects Drp1 mitochondrial recruitment and fission. Here, we used *in vivo* chemical crosslinking to explore the self-assembly state of Drp1 and how it regulates the association of Drp1 with MIEFs and Mff. We show that in intact mammalian cells Drp1 exists as a mixture of multiple self-assembly forms ranging from the minimal, probably tetrameric, self-assembly subunit to several higher order oligomers. Precluding mitochondria-bound Drp1 in Mff/MIEF1/2-deficient cells does not affect the oligomerization state of Drp1, while conversely forced recruitment of Drp1 to mitochondria by MIEFs or Mff facilitates Drp1 oligomerization. Mff preferentially binds to higher order oligomers of Drp1, whereas MIEFs bind to a wider-range of Drp1 assembly subunits, including both lower and higher oligomeric states. Mff only recruits active forms of Drp1, while MIEFs are less selective and recruit both active and inactive Drp1 as well as oligomerization- or GTPase-deficient Drp1 mutants to mitochondria. Moreover, all the fission-incompetent Drp1 mutants tested (except the monomeric mutant K668E) affect Drp1-driven mitochondrial dynamics via incorporation of the mutants into the native oligomers to form function-deficient Drp1 assemblies. We here confirm that MIEFs also serve as a platform facilitating the binding of Drp1 to Mff and loss of MIEFs severely impairs the interaction between Drp1 and Mff. Collectively, our findings suggest that Mff and MIEFs respond differently to the molecular assembly state of Drp1 and that the extent of Drp1 oligomerization regulates mitochondrial dynamics.

## Introduction

Mitochondria are highly dynamic organelles that frequently change their shapes by shifting the balance between fission and fusion events in response to various cellular conditions. The opposing processes of mitochondrial fission and fusion are mediated by several evolutionarily conserved large dynamin superfamily GTPases (Pagliuso et al., 2018; Tilokani et al., 2018; Giacomello et al., 2020; Yu et al., 2020). Three mitochondrial membrane-anchored dynamin-related proteins, mitofusins (Mfn1 and Mfn2) that reside in the mitochondrial outer membrane (MOM) and OPA1 that is localized in the mitochondrial inner membrane (MIM), are responsible for fusion of the mitochondrial outer and inner membranes. Mitochondrial fission is mediated by the highly conserved dynamin-related GTPase Drp1. In mammalian cells, Drp1 primarily resides in the cytosol, but can be recruited to mitochondria through its mitochondrial receptors, Mff and MIEFs (MIEF1/MiD51 and MIEF2/MiD49), which are anchored in the mitochondrial outer membrane (Otera et al., 2010; Palmer et al., 2011; Zhao et al., 2011; Liu et al., 2013; Yu et al., 2017). Drp1 recruitment to mitochondria by Mff and MIEFs however results in different outcomes: overexpression of Mff promotes fission leading to mitochondrial fragmentation, whereas overexpression of MIEF1 or MIEF2 inhibits fission resulting in mitochondrial elongation (Otera et al., 2010; Zhao et al., 2011; Liu et al., 2013; Loson et al., 2013).

In addition to the crucial roles of Mff and MIEFs in the Drp1 recruitment process, emerging evidence suggests that the molecular assembly state of Drp1 also plays a role in mitochondrial recruitment (Zhu et al., 2004; Palmer et al., 2011; Liu and Chan, 2015; Clinton et al., 2016). Drp1 can self-assemble into higher order complexes that mediate mitochondrial fission. *In vitro* studies have suggested that mammalian Drp1 in solution exists in multiple assembly states, probably including dimers, tetramers, and higher order oligomers in a dynamic equilibrium (Shin et al., 1999; Zhu et al., 2004; Frohlich et al., 2013; Macdonald et al., 2014; Ugarte-Uribe et al., 2014; Liu and Chan, 2015; Hatch et al., 2016). Whether this is the case in intact cells is however poorly understood, and data proposing dimers (Koirala et al., 2013; Macdonald et al., 2014), tetramers (Shin et al., 1999; Zhu et al., 2004; Bossy et al., 2010; Ugarte-Uribe et al., 2014) or a dynamic dimer-tetramer equilibrium (Chang et al., 2010; Frohlich et al., 2013; Liu and Chan, 2015) have been presented. In addition, how these different assembly subunits of Drp1 regulate its mitochondrial recruitment through the interaction with Mff and MIEFs remains to be elucidated (Liu and Chan, 2015; Clinton et al., 2016).

In this report, we demonstrate that human Drp1 exists in intact cells in different oligomerization states, starting from a probably tetrameric form and proceeding to several higher order subunits in a dynamic equilibrium. We reveal differences in how oligomeric states are recognized by Mff and MIEFs: MIEFs interact with both active and inactive Drp1 forms, while Mff interacts only with active forms. Furthermore, Mff preferentially binds to and recruits higher-order oligomeric forms of Drp1 to mitochondria, whereas MIEFs bind to a wider-range of Drp1 oligomeric subunits and compile them into ring-like structures on mitochondria regardless of whether Drp1 is in its active state or not. Interestingly, all the fission-incompetent Drp1 mutants (except the monomeric mutant K668E) can affect Drp1-driven mitochondrial dynamics via incorporating the mutants into the native oligomers to form functionally deficient Drp1 assemblies. Our data suggest that the molecular state of Drp1 is crucial for its selective recruitment to mitochondria by MIEFs or Mff and is an important regulator of mitochondrial dynamics. Thus, manipulation of Drp1 molecular state may be a potential therapeutic target for treating various mitochondrial diseases.

## Results

### The Minimal Self-Assembly Subunit of Drp1 in Intact Mammalian Cells Is Probably a Tetramer

**FIGURE 1:**
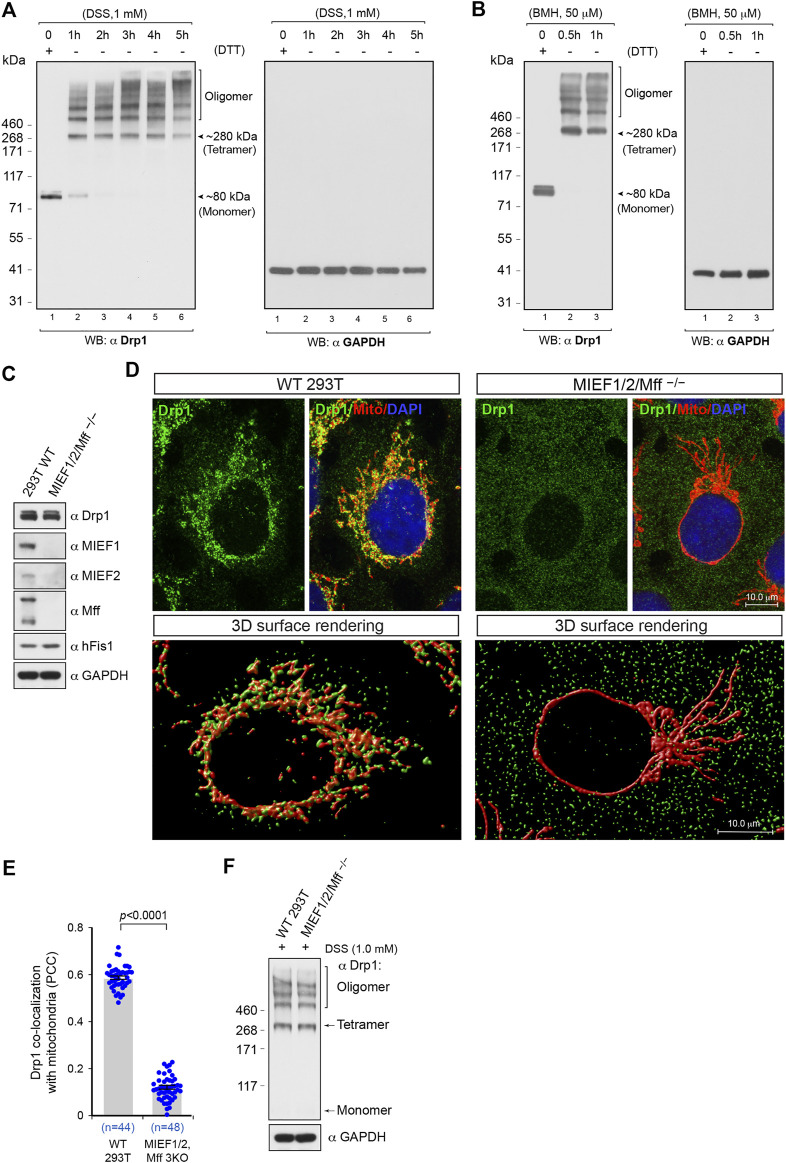
The oligomeric state of Drp1 in human (293T) cells in vivo and the receptor-mediated recruitment of Drp1 to mitochondria enhances Drp1 oligomerization. **(A,B)** In 293T cells, Drp1 exists as multiple self-assembly subunits including the minimal self-assembly unit at ∼280 kDa (probably corresponding to tetramers) and several higher order units. 293T cells were treated with the cell-permeable crosslinking reagent DSS **(A)** or BMH **(B)** in a time course as indicated. Cell lysates were analyzed by Western blotting as indicated. The endogenous monomeric protein GAPDH was used as negative control to rule out nonspecific crosslinking. **(C)** The single cell derived colony of MIEF1/2/Mff^−/−^ 293T cells (triple receptor knock-out) was validated by Western blotting analysis with indicated antibodies. WT 293T cells were used as control. **(D)** Confocal images and 3D surface rendering of mitochondrial morphology and endogenous Drp1 distribution in WT and MIEF1/2/Mff^−/−^ 293T cells. Cells growing on glass coverslips were stained with MitoTracker (red) before fixation, followed by immunostaining with anti-Drp1 antibody (green). The cell nuclei were stained by DAPI (blue) **(upper panels)**. 3D Surface rendering of confocal images with Drp1 (green) and mitochondria (red) in WT **(lower left panel)** and MIEF1/2/Mff^−/−^ 293T cells **(lower right panel)**. **(E)** Quantitative co-localization of endogenous Drp1 with mitochondria in **(D)** was analyzed by the *PCC* (mean ± S.E.M.). n represents the number of cells analyzed. **(F)** The oligomeric states of endogenous Drp1 in WT or MIEF1/2/Mff^−/−^ 293T cells. Cultured cells were treated with freshly-prepared 1 mM DSS for 3 h at room temperature, followed by immunoblotting with indicated antibodies.

We were first interested in assessing the oligomerization state of Drp1 in intact 293T cells. To this end, we conducted *in vivo* chemical crosslinking using the cell-permeable crosslinker disuccinimidyl suberate (DSS) to capture native Drp1 self-assembly subunits as previously described (Dettmer et al., 2013) in a time course experiment. In cells not treated with DSS, endogenous Drp1 was observed by SDS-PAGE under DTT-reducing conditions as a single band with a molecular weight at ∼80 kDa, corresponding to the Drp1 monomer (Figure 1A, left panel, lane 1). Upon treatment with DSS (1 mM), the monomeric form of Drp1 gradually disappeared in a time-dependent manner, and multiple higher assembly units of Drp1 were observed as bands ranging from a species migrating at an apparent molecular weight of ∼280 kDa to several higher order oligomers (Figure 1A, see also Supplementary Figure S1A). The ∼280 kDa species, although an approximation of the real molecular weight (Rath et al., 2009; Shirai et al., 2008), likely corresponds to the tetrameric form, in line with previous publications (Shin et al., 1999; Zhu et al., 2004). Previous studies have suggested that the minimal self-assembly subunit of Drp1 in intact cells may be a dimer (Koirala et al., 2013; Macdonald et al., 2014), tetramer (Shin et al., 1999; Zhu et al., 2004; Bossy et al., 2010; Ugarte-Uribe et al., 2014) or a dynamic dimer-tetramer equilibrium (Chang et al., 2010; Frohlich et al., 2013; Liu and Chan, 2015) but not a monomer. A similar Drp1 band pattern was observed also after treatment with another *in vivo* crosslinking reagent bismaleimidohexane (BMH) (Figure 1B, left panel). Furthermore, to avoid potential artifacts introduced by chemical crosslinking, we used GAPDH as a negative control. The migration of GAPDH was not affected by *in vivo* crosslinking treatment with DSS or BMH at different time points (Figures 1A,B, right panels), as previously reported (Li et al., 2015), indicating that *in vivo* chemical crosslinking with DSS or BMH does not result in non-specific crosslinking of cellular proteins into artificial oligomers. Moreover, no MIEF1/2 or Mff bands larger than 250 kDa were observed after *in vivo* crosslinking treatment (Supplementary Figures S1B–D), indicating that MIEFs and Mff do not affect the Drp1 oligomeric state. As the time-course experiments showed that treatment of living cells with 1 mM DSS for 3 h was sufficient to keep native Drp1 complexes in different assembly forms, we therefore used this regiment (1 mM DSS for 3 h) for evaluating the native oligomeric state of different Drp1 mutants in subsequent experiments.

### Receptor-Mediated Mitochondrial Recruitment of Drp1 Enhances Drp1 Oligomerization

The data above delineate the oligomerization state of the total pool of Drp1 in the cell, and we next evaluated the assembly profile of Drp1 in the cytosol *in vivo*, when mitochondrial recruitment was inhibited. To accomplish this, we generated triple knockout 293T cells lacking the mitochondrial receptors MIEF1, MIEF2, and Mff (MIEF1/2/Mff^−/−^) by CRISPR/Cas9-mediated genome editing as confirmed by Western blotting (Figure 1C). In the MIEF1/2/Mff^−/−^ 293T cells, mitochondria exhibited a super-fused network, and Drp1 was diffusely distributed in the whole cytosol, resulting in a prominent decrease of Drp1 puncta on mitochondria (Figure 1D, upper right panel) compared to WT 293T cells (Figure 1D, upper left panel). Ablation of both MIEFs and Mff led to a severe loss of mitochondrially-bound Drp1 punctate structures as displayed by 3D surface rendering of confocal images (Figure 1D, lower right panel) compared with those in WT 293T cells (Figure 1D, lower left panel). Co-localization analysis by the Pearson’s correlation coefficient (*PCC*) further indicated that the triple knockout of MIEF1/2 and Mff significantly reduced the amount of Drp1 on mitochondria (Figure 1E). *In vivo* chemical crosslinking (1 mM DSS for 3 h) followed by immunoblotting showed that shifting Drp1 from mitochondria to the cytosol did not significantly alter the equilibrium of the Drp1 oligomeric state in MIEF1/2/Mff^−/−^ 293T cells as compared to WT 293T cells (Figure 1F).

**FIGURE 2:**
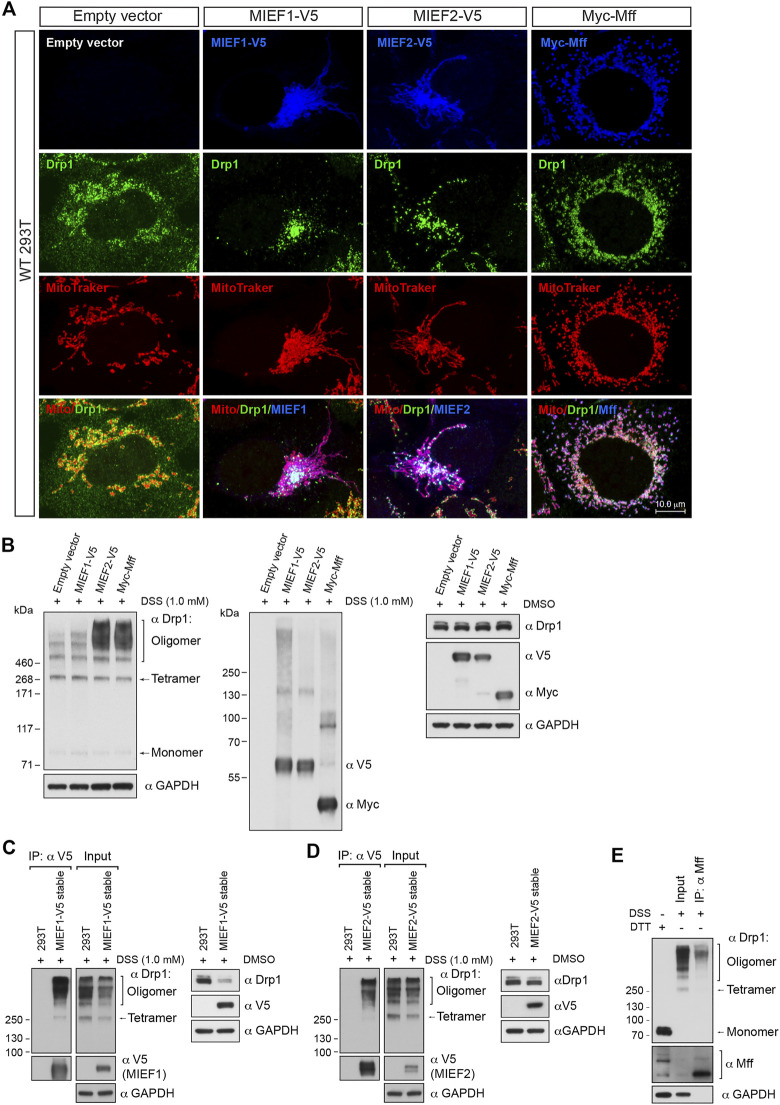
Receptor-mediated mitochondrial recruitment of Drp1 enhances Drp1 oligomerization. **(A)** Confocal images of mitochondrial morphology and Drp1 distribution in WT 293T cells transfected with empty vector, MIEF1-V5, MIEF2-V5, or Myc-Mff. At 16 h post-transfection, cells were stained with MitoTracker (red) before fixation, followed by immunostaining with anti-Drp1 (green) and anti-V5 or anti-Myc (blue) antibodies (white color indicates co-localization of mitochondria, Drp1 and the respective receptor protein). **(B)** The oligomeric states of Drp1 in WT 293T cells transfected with empty vector, MIEF1-V5, MIEF2-V5, or Myc-Mff. 293T cells were transfected with indicated plasmids for 18 h and incubated with freshly-prepared 1 mM DSS or DMSO (control) for 3 h at room temperature, followed by Western blotting with indicated antibodies. **(C,D)** MIEF1/2 interact with a wider-range of Drp1 assembly subunits. WT 293T cells and 293T cell lines stably expressing MIEF1-V5 **(C)** or MIEF2-V5 **(D)** were treated with freshly-prepared 1 mM DSS or DMSO (control) for 3 h at room temperature and subjected to co-IP with anti-V5 beads. The immunoprecipitates were analyzed by immunoblotting with indicated antibodies. **(E)** Mff preferentially binds to higher order oligomers of Drp1. WT 293T cells were treated with freshly-prepared 1 mM DSS for 3 h at room temperature and subjected to co-IP. Cell lysates were incubated with Dynabeads™ protein G beads pre-conjugated with 2 μg anti-Mff antibody overnight at 4°C. The bead-binding proteins were dissolved and analyzed by Western blotting with indicated antibodies.

In a converse experiment, we overexpressed Mff or MIEFs in order to enhance recruitment of Drp1 to the mitochondria. Elevated levels of MIEF1, MIEF2 or Mff led to recruitment of most of the cytosolic Drp1 to mitochondria, in the form of punctuate structures (Figure 2A). *In vivo* chemical crosslinking followed by immunoblotting revealed that Mff-, MIEF1-, and especially MIEF2-mediated mitochondrial accumulation of Drp1 affected the oligomeric state of Drp1 in cells, facilitating Drp1 oligomerization (Figure 2B). In summary, our data suggest that Drp1 exists as a mixture of multiple oligomeric forms including tetramers and several higher order oligomers in the cytosolic pool, whereas receptor-mediated mitochondrial recruitment leads to a shift towards higher order oligomers of Drp1. Furthermore, increasing recruitment of Drp1 to mitochondria shifted the equilibrium of the Drp1 oligomeric state towards higher-order oligomerization *in vivo*.

### Mff Preferentially Binds to Higher Order Oligomers of Drp1 While MIEFs Bind to a Wider Range of Drp1 Assembly Forms

The data presented above indicate that receptor-mediated mitochondrial recruitment enhances Drp1 higher order oligomerization. We next evaluated the oligomeric state of endogenous Drp1 bound to MIEFs and Mff by *in vivo* chemical crosslinking followed by co-immunoprecipitation (co-IP). Due to the low levels of endogenous MIEFs in 293T cells, we in this set of experiments used 293T cell lines with stable expression of MIEF1-V5 or MIEF2-V5 (Yu et al., 2017). Following cross-linking with DSS (1 mM for 3 h), co-IP experiments for Mff or MIEFs were conducted. This revealed that MIEF1 and MIEF2 did bind to a wider range of both lower and higher Drp1 oligomerization forms but with an increased association to higher-order oligomers of Drp1 (Figures 2C,D). In contrast, endogenous Mff preferentially bound higher order oligomers of Drp1 (Figure 2E). Collectively, these results suggest that MIEFs bind to a broader spectrum of Drp1 oligomerization forms, whereas Mff mostly binds to higher order oligomers of Drp1.

### The Self-Assembly State of Drp1 Regulates its Binding to the Mitochondrial Receptors MIEFs and Mff

**FIGURE 3:**
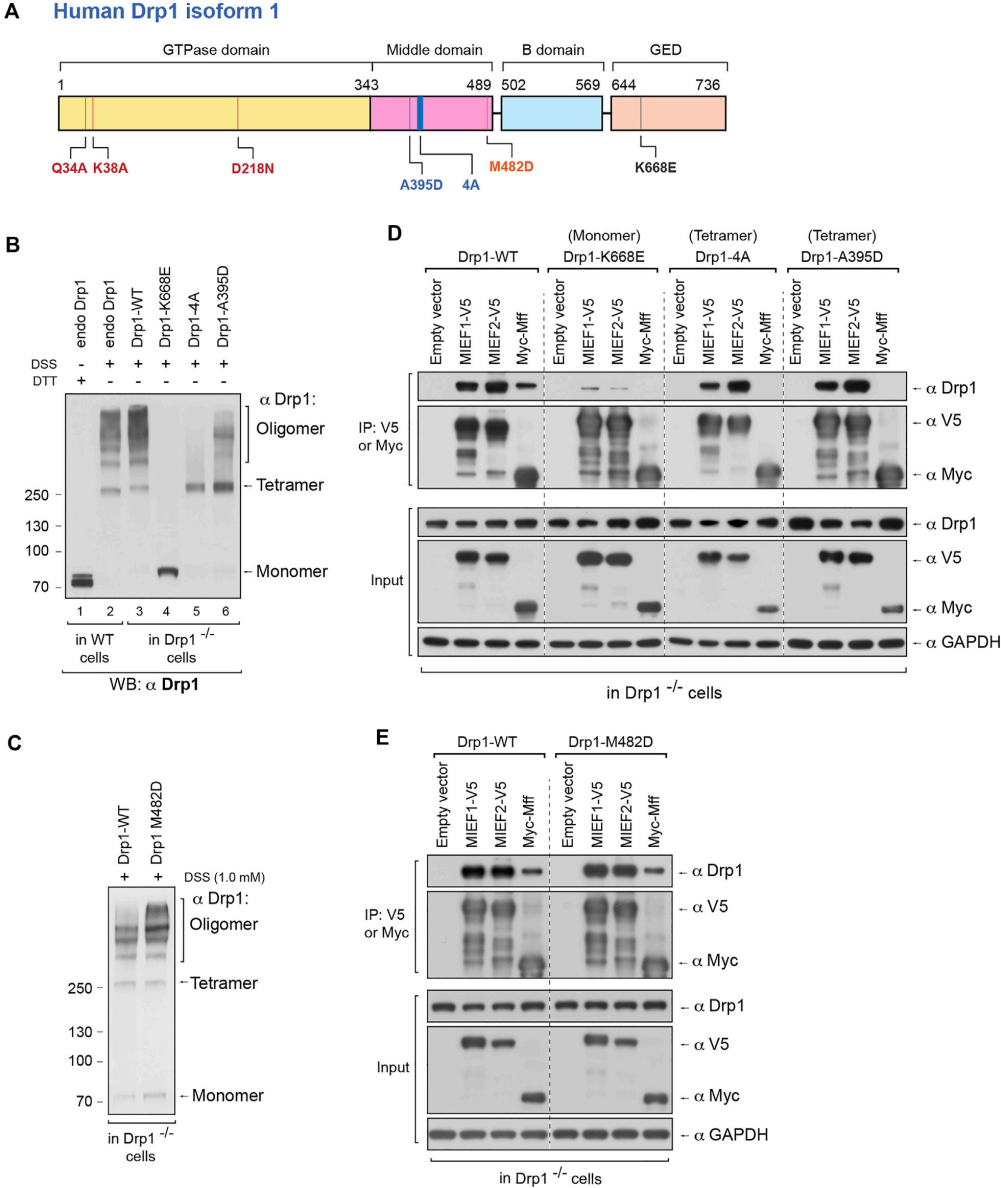
The self-assembly state of Drp1 differentially regulates its binding to the mitochondrial receptors MIEFs and Mff. **(A)** Schematic representation of human Drp1 protein structure and functional domains. The positions of Drp1 mutations used in this study are indicated. **(B)** The oligomeric states of Drp1-WT, Drp1-K668E, Drp1-4A, and Drp1-A395D. Drp1^−/−^ 293T cells were transfected with untagged wild-type Drp1 (Drp1-WT) or untagged Drp1 mutants as indicated for 18 h, treated with *in vivo* chemical crosslinker DSS (1 mM) for 3 h and analyzed by Western blotting with anti-Drp1 antibody. Endogenous Drp1 in wild-type 293T cells (endo Drp1) and exogenous Drp1-WT in Drp1^−/−^ 293T cells were used as controls. **(C)** The oligomeric state of Drp1-M482D. Drp1^−/−^ 293T cells were transfected with untagged Drp1-WT (control) or Drp1-M482D as indicated for 18 h and incubated with DSS (1 mM) for 3 h at room temperature, followed by Western blotting with anti-Drp1 antibody. **(D)** The Drp1 oligomerization-defective mutants Drp1-K668E, Drp1-4A, and Drp1-A395D interact differently with MIEFs and Mff. Drp1^−/−^ 293T cells were co-transfected with indicated plasmids. Cell lysates were used for co-IP with anti-V5 or anti-Myc agarose beads as indicated, and the immunoprecipitates were analyzed by Western blotting with indicated antibodies. **(E)** The dominant oligomeric mutant Drp1-M482D shows intact binding with MIEFs and Mff. Drp1^−/−^ 293T cells were co-transfected with indicated plasmids and subjected to co-IP. Cell lysates were incubated with anti-V5 or anti-Myc agarose beads as indicated. The proteins associated with MIEFs or Mff in immunoprecipitates were detected by Western blotting with indicated antibodies.

We were next interested in exploring how perturbing the oligomerization capability of Drp1 would affect the choice of recruitment receptors for Drp1. A number of Drp1 mutants have been identified, which either reduce or enhance oligomerization of Drp1. The Drp1-K668E mutation (in human Drp1 isoform 1, corresponding to K642E in human isoform 3) has been shown to produce a monomeric form of Drp1 in solution (Frohlich et al., 2013). Drp1-A395D is a disease-associated lethal mutation (Waterham et al., 2007) generating a Drp1 with defects in higher order assembly (Chang et al., 2010). Furthermore, the Drp1-4A mutant (the four residues 401GPRP404 mutated to AAAA in human Drp1 isoform 1) only forms dimers but not higher order oligomers in solution (Frohlich et al., 2013). Conversely, the Drp1-M482D mutant yields a Drp1 with an increased higher oligomerization level (Frohlich et al., 2013). All these mutations in the Drp1 protein are depicted in Figure 3A. The expression levels of Drp1 WT and the various Drp1 mutants in Drp1-deficient 293T cells (Drp1^−/−^ 293T cells) are shown in Supplementary Figure S2B.

To assess the effects of these Drp1 mutations on mitochondrial recruitment and recruitment receptor preference, we used Drp1^−/−^ 293T cells as a model (Yu et al., 2019), to avoid interference of endogenous Drp1. Meanwhile, in order to exclude any effect of immunotags on Drp1 structure and function (Montecinos-Franjola et al., 2020), untagged Drp1-WT, Drp1-K668E, Drp1-A395D, Drp1-4A, and Drp1-M482D mutants were expressed in the Drp1^−/−^ 293T cells. *In vivo* chemical crosslinking revealed that exogenous Drp1-WT in Drp1^−/−^ 293T cells as well as endogenous Drp1 in WT 293T cells formed multiple assembly subunits with a minimal species at ∼280 kDa (Figure 3B, lanes 2 and 3). In contrast, the K668E mutant only yielded a monomeric band at ∼80 kDa (Figure 3B, lane 4), in line with a previous report that Drp1-K668E exists as a monomer in solution (Frohlich et al., 2013). The Drp1-4A mutant resulted in a crosslinked product at ∼280 kDa (corresponding to tetramers), but did not generate higher order oligomers (Figure 3B, lane 5). This is in contrast to a previous report, showing that Drp1-4A produced only dimers in solution (Frohlich et al., 2013). Similarly, the Drp1-A395D mutant exhibited a major cross-linked species at ∼280 kDa whereas higher order assembly was severely impaired (Figure 3B, lane 6), consistent with a previous report (Chang et al., 2010). Expression of the oligomerization-promoting Drp1-M482D mutant resulted in an increase of higher-order oligomers in Drp1^−/−^ 293T cells compared to expression of Drp1-WT (Figure 3C). In summary, our *in vivo* crosslinking data indicate that the K668E mutant is a monomer with a complete loss of Drp1 self-assembling ability, whereas the Drp1-4A and A395D mutants mainly form tetramers (at ∼280 kDa) with defective higher order assembly of Drp1 in intact cells. In contrast, the M482D mutant enhanced the formation of higher order Drp1 complexes.

We next evaluated the ability of the oligomerization-deficient or -promoting Drp1 mutants to interact with MIEFs and Mff by co-immunoprecipitation (co-IP). The Drp1-4A and A395D mutants were able to bind MIEF1 and MIEF2 as efficiently as WT Drp1 (Drp1-WT), but the monomeric mutant K668E showed profoundly impaired binding to MIEF1 and MIEF2 compared with wild-type Drp1 (Figure 3D). All three oligomerization-deficient Drp1 mutants (Drp1-4A, A395D, and K668E) were unable to bind Mff at all (Figure 3D). In contrast, the oligomerization-promoting M482D mutant robustly interacted with both MIEFs and Mff in Drp1^−/−^ cells, at a level comparable to Drp1-WT (Figure 3E). In summary, these data reveal that Drp1 mutants with impaired oligomerization potential show selectivity with regard to receptor binding, while an oligomerization-promoting mutant can interact with both Mff and MIEFs.

### The Self-Assembly State of Drp1 Differentially Affects Its Mitochondrial Recruitment Through MIEFs or Mff

**FIGURE 4:**
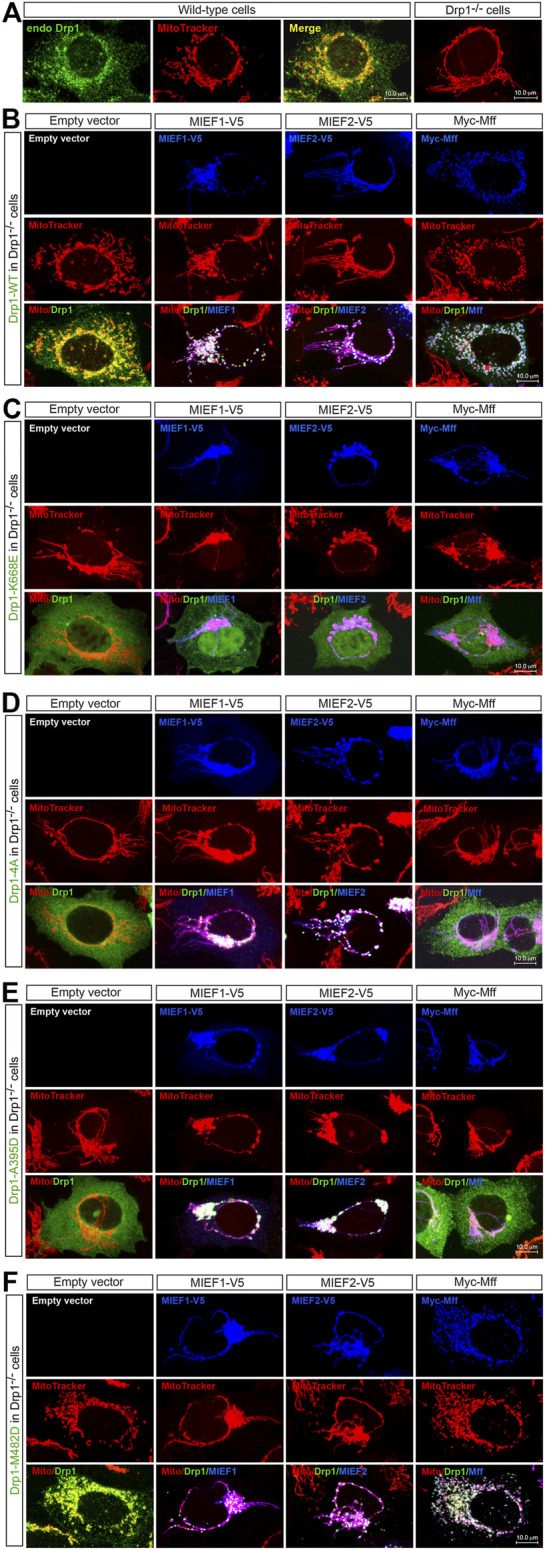
The self-assembly state of Drp1 differentially regulates its mitochondrial recruitment through MIEFs or Mff **(A)** Confocal images of mitochondrial morphology and endogenous Drp1 distribution in wild-type (WT) or Drp1^−/−^ 293T cells stained with MitoTracker (red) and anti-Drp1 (green) antibody. **(B–F)** Confocal images of mitochondrial morphology and exogenous Drp1 distribution in Drp1^−/−^ 293T cells co-transfected with Drp1-WT or an indicated Drp1 mutant and empty vector, MIEF1-V5, MIEF2-V5 or Myc-Mff. At 16 h post-transfection, cells were stained with MitoTracker (red) before fixation, followed by immunostaining with anti-Drp1 (green) and anti-V5 or anti-Myc (blue) antibodies. (Yellow color indicates co-localization of Drp1 with mitochondria. White color indicates co-localization of Drp1 and the respective receptor protein on mitochondria.)

We next analyzed the subcellular distribution of the oligomerization-deficient and -promoting Drp1 mutants in relation to different levels of Mff and MIEFs. Like endogenous Drp1 in WT 293T cells (Figure 4A, left panels), exogenous Drp1-WT was distributed in both the cytosol and on mitochondria in Drp1^−/−^ cells (Figure 4B, left panel), and co-expression of MIEF1, MIEF2 or Mff promoted recruitment of Drp1-WT from the cytosol to mitochondria (Figure 4B, middle and right panels). In contrast, all the three oligomerization-deficient Drp1 mutants (K668E, Drp1-4A, and A395D) were diffusely distributed in the cytosol when expressed in Drp1^−/−^ cells alone (Figures 4C–E, left panels). Exogenous expression of MIEF1 or MIEF2 efficiently recruited Drp1-4A and A395D but not K668E from the cytosol to mitochondria (Figures 4C–E, middle panels). However, increasing Mff expression did not change the cytosolic distribution of the three oligomerization-deficient Drp1 mutants (Figures 4C–E, right panels), in line with our results from co-IP (Figure 3D). Interestingly, Drp1-M482D was distributed in both the cytosol and on mitochondria in Drp1^−/−^ 293T cells (Figure 4F, left panels) in a manner similar to that seen following exogenous expression of Drp1-WT in Drp1^−/−^ cells (see Figure 4B, left panels). Co-expression of MIEF1, MIEF2 or Mff further recruited Drp1-M482D to mitochondria in Drp1^−/−^ cells (Figure 4F, middle and right panels), comparable to Drp1-WT recruited by MIEFs or Mff in Drp1^−/−^ cells (see Figure 4B, middle and right panels).

Collectively, these data indicate that oligomerization-deficient Drp1 mutants lose their ability to interact with Mff and only interact with MIEFs, while an oligomerization-promoting Drp1 mutant interacts with both Mff and MIEFs. In line with this, exogenous expression of Mff and MIEFs enhanced mitochondrial recruitment of the oligomerization-promoting mutant, while only MIEFs, but not Mff, recruited the oligomerization-deficient forms of Drp1 to mitochondria.

### GTPase-Deficient Drp1 Mutants Are Recruited to Mitochondria Through MIEF1/2 but Not Through Mff

**FIGURE 5:**
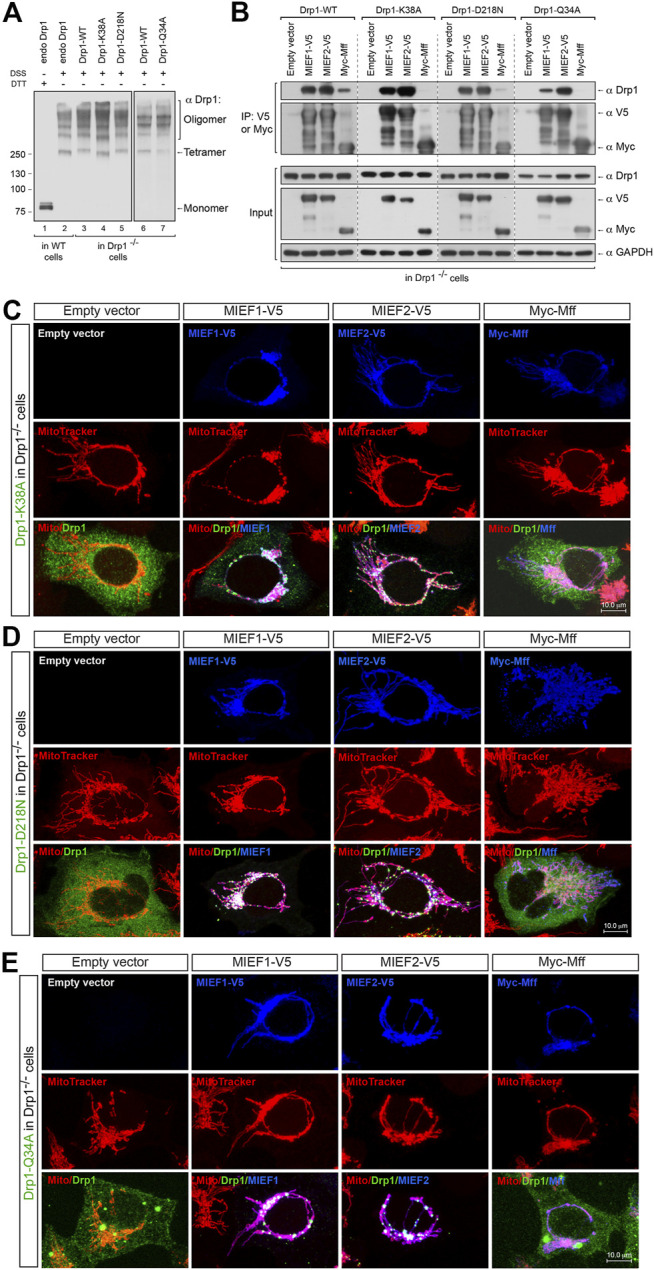
MIEF1/2 but not Mff interact with and recruit the GTPase-defective mutants to the mitochondrial surface. **(A)** The oligomeric states of Drp1-K38A, Drp1-D218N, and Drp1-Q34A. Drp1^−/−^ 293T cells were transfected with untagged Drp1-WT, Drp1-K38A, Drp1-D218N or Drp1-Q34A as indicated for 18 h, treated with *in vivo* chemical crosslinker DSS (1 mM) for 3 h at room temperature and analyzed by Western blotting with anti-Drp1 antibody. Endogenous Drp1 (endo Drp1) in wild-type 293T and exogenous Drp1-WT in Drp1^−/−^ 293T cells were used as controls. **(B)** Effects of the GTPase-defective mutants Drp1-K38A, Drp1-D218N, and Drp1-Q34A on the association of Drp1 with MIEFs and Mff. Drp1^−/−^ 293T cells were co-transfected with indicated plasmids and subjected to co-IP. Cell lysates were incubated with anti-V5 or anti-Myc agarose beads as indicated. The immunoprecipitates were subjected to Western blotting analysis with indicated antibodies. Exogenous Drp1-WT expressed in Drp1^−/−^ 293T cells was used as controls. **(C–E)** Confocal images of mitochondrial morphology and distribution of Drp1-K38A **(C)**, Drp1-D218N **(D)** or Drp1-Q34A **(E)** in Drp1^−/−^ 293T cells co-transfected with an indicated Drp1 mutant and either empty vector, MIEF1-V5, MIEF2-V5 or Myc-Mff. At 16 h post-transfection, cells were stained with MitoTracker (red) before fixation, followed by immunostaining with anti-Drp1 (green) and anti-V5 or anti-Myc (blue) antibodies. (Yellow color indicates co-localization of Drp1 with mitochondria. White color indicates co-localization of Drp1 and the respective receptor protein on mitochondria.)

Besides the importance of Drp1 oligomerization in regulating its binding to MIEFs and Mff, the GTPase activity of Drp1 also plays a vital role in these processes. It is believed that Drp1 GTP binding, hydrolysis and release result in its association and disassociation with MIEFs and Mff on the mitochondrial outer membrane in the fission process. To specifically address this, we investigated how the GTPase-deficient Drp1 mutants Drp1-K38A, Drp1-D218N, and Drp1-Q34A (see Figure 3A) bound to Mff and MIEFs. Drp1-K38A binds to but does not hydrolyze or release GTP, thus locking Drp1 in the GTP-bound state and exhibiting a complete loss of GTPase activity (Yoon et al., 2001; Zhu et al., 2004). Drp1-D218N (a GTP-binding-defective mutant equivalent to the D231N in rat Drp1) as well as Drp1-Q34A was reported to lack all GTPase activity (Yoon et al., 2001; Wenger et al., 2013). *In vivo* chemical crosslinking followed by immunoblotting showed that untagged Drp1-K38A (in Drp1 isoform 3), Drp1-D218N and Drp1-Q34A (in Drp1 isoform 1) were oligomerized with a similar pattern as endogenous Drp1 seen in WT 293T cells and as exogenous Drp1-WT when expressed in Drp1^−/−^ cells (Figure 5A), implying that GTPase-deficient mutants do not have impaired self-assembly of Drp1 in cells. Co-IP showed that the three GTPase-deficient mutants efficiently bound to MIEF1 and MIEF2, whereas interaction with Mff was severely impaired (Figure 5B). Consistent with this, immunofluorescence showed that Drp1-K38A, Drp1-D218N, and Drp1-Q34A mutants were mainly cytosolic in Drp1^−/−^ cells (Figures 5C–E, left panels), but were recruited to mitochondria by increasing levels of MIEF1 or MIEF2, while Mff did not enhance mitochondrial recruitment (Figures 5C–E). Together, these data indicate that Mff- but not MIEF-mediated recruitment of Drp1 to mitochondria closely depends on functional Drp1 GTP binding, hydrolysis and release. In summary, all the function-deficient Drp1 mutants analyzed here except the monomeric mutant K668E display a severely decreased interaction with Mff, but retain normal interaction with MIEF1 and MIEF2.

### The Oligomerization-Promoting Mutant Drp1-M482D Is More Competent Than Drp1-WT in Mitochondrial Fission

**FIGURE 6:**
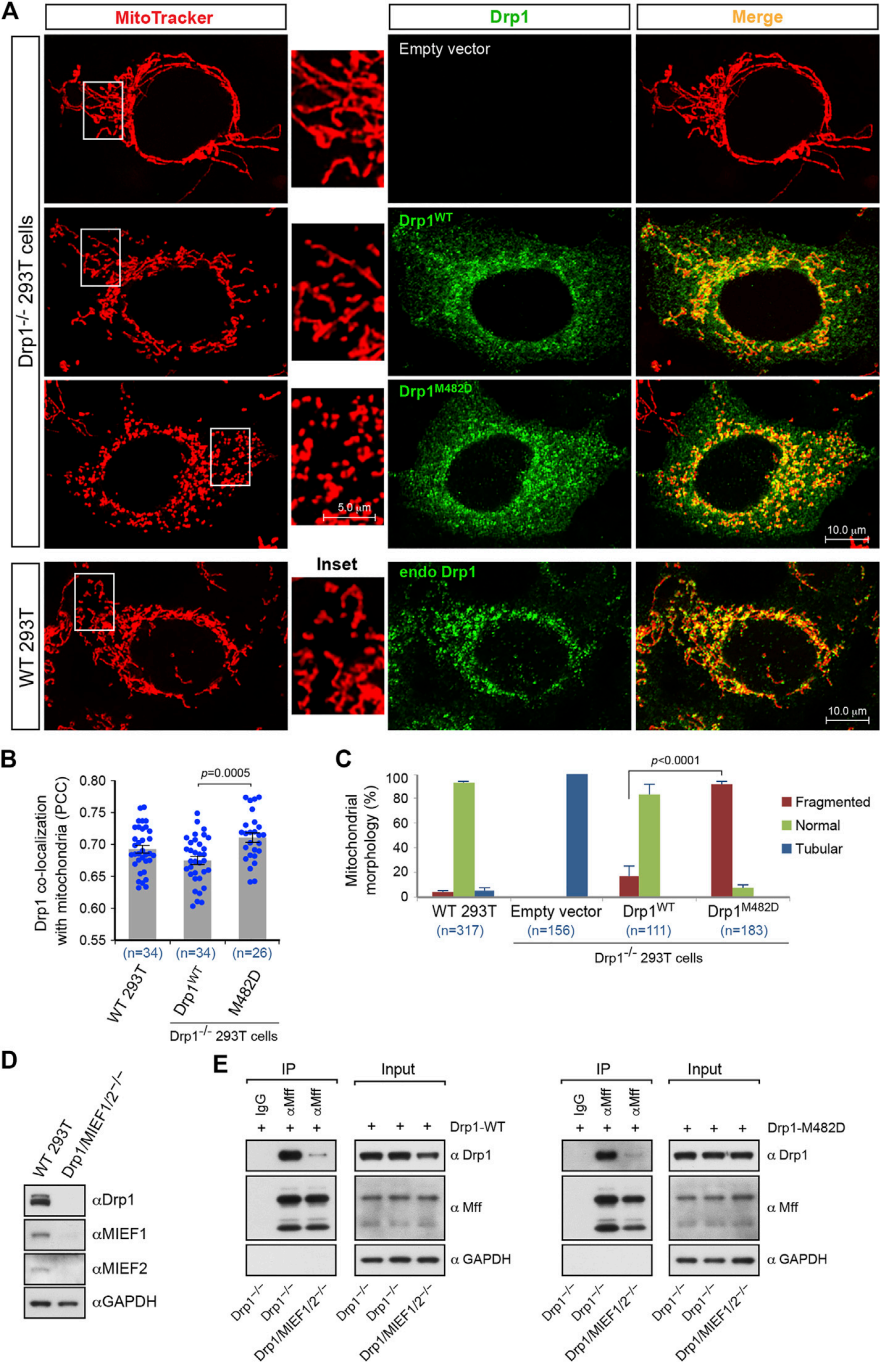
The dominant oligomeric mutant Drp1-M482D is more competent in inducing mitochondrial fission than Drp1-WT. **(A)** Confocal images of mitochondrial morphology and Drp1 distribution in WT 293T cells and Drp1^−/−^ 293T cells transfected with empty vector, Drp1-WT or Drp1-M482D as indicated. At 16 h post-transfection, cells were stained with MitoTracker (red) before fixation, followed by immunostaining with anti-Drp1 antibody (green). Insets represent high magnification views of the boxed areas. **(B)** Quantitative co-localization of Drp1 with mitochondria in (A) was analyzed by the *PCC* (mean ± S.E.M.). n represents the number of cells analyzed. **(C)** Percentages (mean ± S.E.M.) of cells with indicated mitochondrial morphologies in WT 293T, Drp1^−/−^ 293T, and Drp1^−/−^ 293T cells transfected with Drp1-WT or Drp1-M482D as shown in **(A)**. n represents the number of cells analyzed. **(D)** The single cell derived colony of Drp1/MIEF1/2^−/−^ 293T cells was validated by Western blotting analysis with indicated antibodies. WT 293T cells were used as control. **(E)** Loss of MIEF1/2 severely reduced the interaction between endogenous Mff and exogenous Drp1-WT or Drp1-M482D. Cell lysates from Drp1^−/−^ or Drp1/MIEF1/2^−/−^ 293T cells transfected with Drp1-WT **(left)** or Drp1-M482D **(right)** were used for co-immunoprecipitation (IP) with Protein G beads conjugated with goat normal IgG (negative control) or goat anti-Mff antibody, followed by Western blotting with indicated antibodies.

The data described above showed that the oligomerization-promoting mutant Drp1-M482D, like wild-type Drp1, is fission-competent and recruited to mitochondria by both MIEFs and Mff. Given the importance of oligomerization for Drp1 function, we therefore compared the biological properties of the oligomerization-promoting mutant Drp1-M482D and Drp1-WT in mitochondrial fission. Exogenous expression of Drp1-M482D exhibited a similar subcellular distribution to that of Drp1-WT in Drp1^−/−^ cells (Figure 6A, middle panels) as well as to that of endogenous **Drp1** in WT 293T cells (Figure 6A, lower panels). As compared to Drp1-WT, quantitative colocalization analysis showed that expression of Drp1-M482D in Drp1^−/−^ cells resulted in a slight, but statistically significant, increase of this protein on mitochondria (Figure 6B), while Drp1-M482D strongly promoted a mitochondrial fission phenotype (Figure 6C). These experiments indicate that the oligomerization-promoting Drp1-M482D mutant is more efficient than wild-type Drp1 in promoting mitochondrial fission.

Our previous studies have suggested that although Mff and MIEFs both are capable of serving as independent receptors for Drp1 recruitment to mitochondria, MIEFs are essential for the interaction between Drp1 and Mff and for Mff-mediated Drp1 recruitment, and lack of MIEFs severely impairs these processes, while in contrast, lack of Mff did not affect the interaction between Drp1 and MIEFs (Yu et al., 2017). Given the importance of MIEFs and Drp1 oligomerization in mitochondrial fission, we next assessed whether the oligomerization-promoting Drp1-M482D mutant would affect Drp1’s interaction with Mff in the presence and absence of MIEFs. To address this, we used the triple knockout 293T cells lacking Drp1, MIEF1, and MIEF2 (Drp1/MIEF1/2^−/−^) generated by CRISPR/Cas9-mediated genome editing as confirmed by Western blotting (Figure 6D). We found that loss of MIEFs profoundly reduced the interaction between Mff and Drp1-M482D in Drp1/MIEF1/2^−/−^ 293T cells (Figure 6E, right panels) in a manner similar to the interaction between Mff and Drp1-WT (Figure 6E, left panels). This result indicates that albeit Mff can independently recruit Drp1, it is clear that MIEFs serve as molecular adaptors between Drp1 and Mff and play a predominant role in Mff-induced Drp1 recruitment, as previously described (Yu et al., 2017). In summary, our data suggest that the oligomerization-promoting Drp1-M482D mutation largely enhances mitochondrial fission but does not change the MIEF-dependency for the interaction between Mff and Drp1.

### MIEFs Recruit Drp1 to Form a Ring-like Structure Around Mitochondria Irrespective of Whatever State Drp1 Is in

**FIGURE 7:**
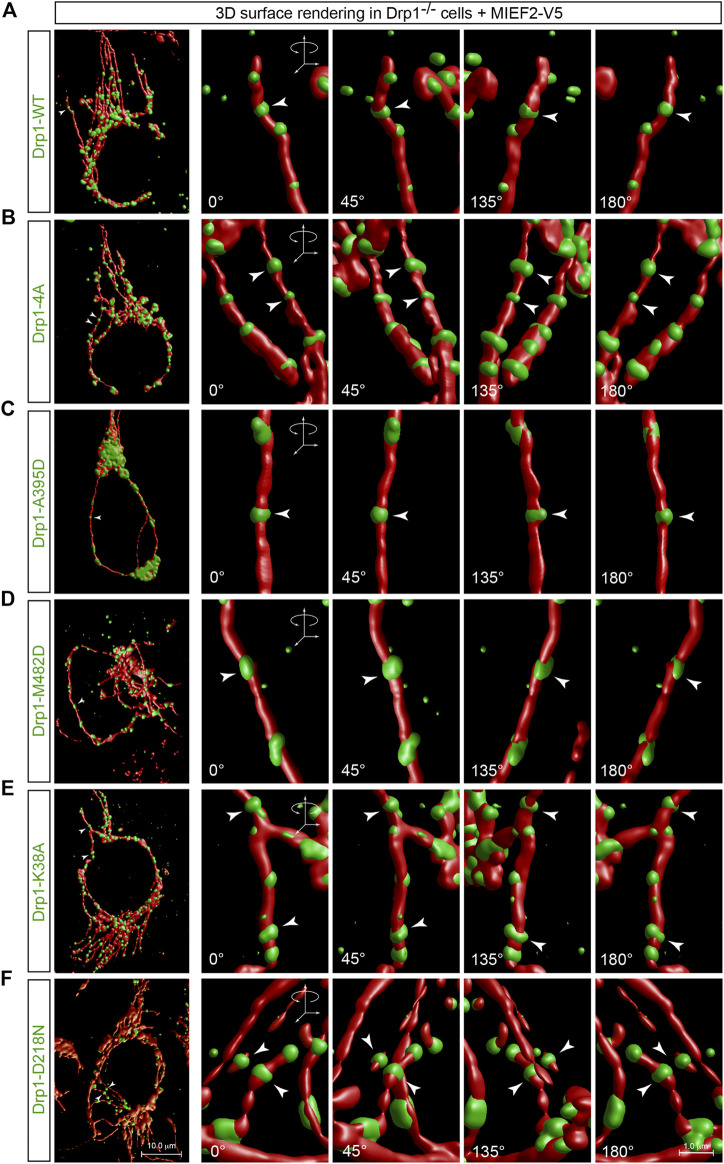
MIEFs induce assembly of Drp1 into ring-like structures wrapping around mitochondria regardless of whether Drp1 is active or not. **(A–F)** 3D surface rendering reconstruction of Drp1 punctate structures on mitochondria in Drp1^−/−^ 293T cells co-transfected with MIEF2-V5 and Drp1-WT **(A)**, Drp1-4A **(B)**, Drp1-A395D **(C)**, Drp1-M482D **(D)**, Drp1-K38A **(E)** or Drp1-D218N **(F)**. At 16 h post-transfection, cells were stained with MitoTracker (red) and anti-Drp1 (green) antibody (the original confocal images are shown in Figures 4, 5). Arrows indicate Drp1 ring-like structures at a potential mitochondrial constriction site.

It is generally thought that in mitochondrial division, the cytosolic Drp1 is recruited to the mitochondrial surface where it assembles into a ring-shaped structure around mitochondria at the potential scission site and ultimately sever the mitochondrion through a GTPase activity-dependent mechanism (Pernas and Scorrano, 2016; Chan, 2020). We were therefore interested in exploring whether the process of assembling Drp1 into a ring-like structure was correlated with its molecular state. To address this issue, we performed 3D surface rendering reconstruction of confocal images with punctate Drp1 structures on mitochondria as recruited by MIEF2 in Drp1^−/−^ cells (see Figures 4, 5). We found that WT Drp1 recruited by MIEF2 could be assembled into a ring-like structure wrapping around mitochondria (Figure 7A, arrowhead; for original images see Figure 4B). Interestingly, a similar ring-shaped structure around mitochondria was also observed when the oligomerization-deficient mutants Drp1-4A and Drp1-A395D as well as the oligomerization-promoting mutant Drp1-M482D were co-expressed with MIEF2 in Drp1^−/−^ cells (Figures 7B–D, arrowheads; for original images see Figures 4D–F). These data indicate that MIEFs cluster Drp1 into ring-like structures regardless of whether Drp1 is in a lower or higher order oligomeric state. Subsequently, we assessed whether GTPase activity was required in this process. We found that like WT Drp1, the GTPase-deficient mutants Drp1-K38A and Drp1-D218N when recruited by MIEF2 were still able to form a ring-shaped structure around mitochondria (Figures 7E,F, arrowheads; for original images see Figures 5C,D). Moreover, we transfected Drp1/MIEF1/2 triple KO cells with Drp1 WT or mutant forms, and performed immunofluorescence staining. The results showed that neither Drp1 oligomerization-deficient nor GTPase-deficient mutants were recruited to mitochondria in the absence of endogenous MIEF1 and MIEF2 (Supplementary Figure S3). Collectively, these observations suggest that Drp1 assembly into a spiral-like structure around mitochondria via MIEFs does not depend on the oligomeric state or the GTPase activity of Drp1.

### The Function-Deficient Drp1 Mutants Counteract Drp1-Mediated Mitochondrial Fission

**FIGURE 8:**
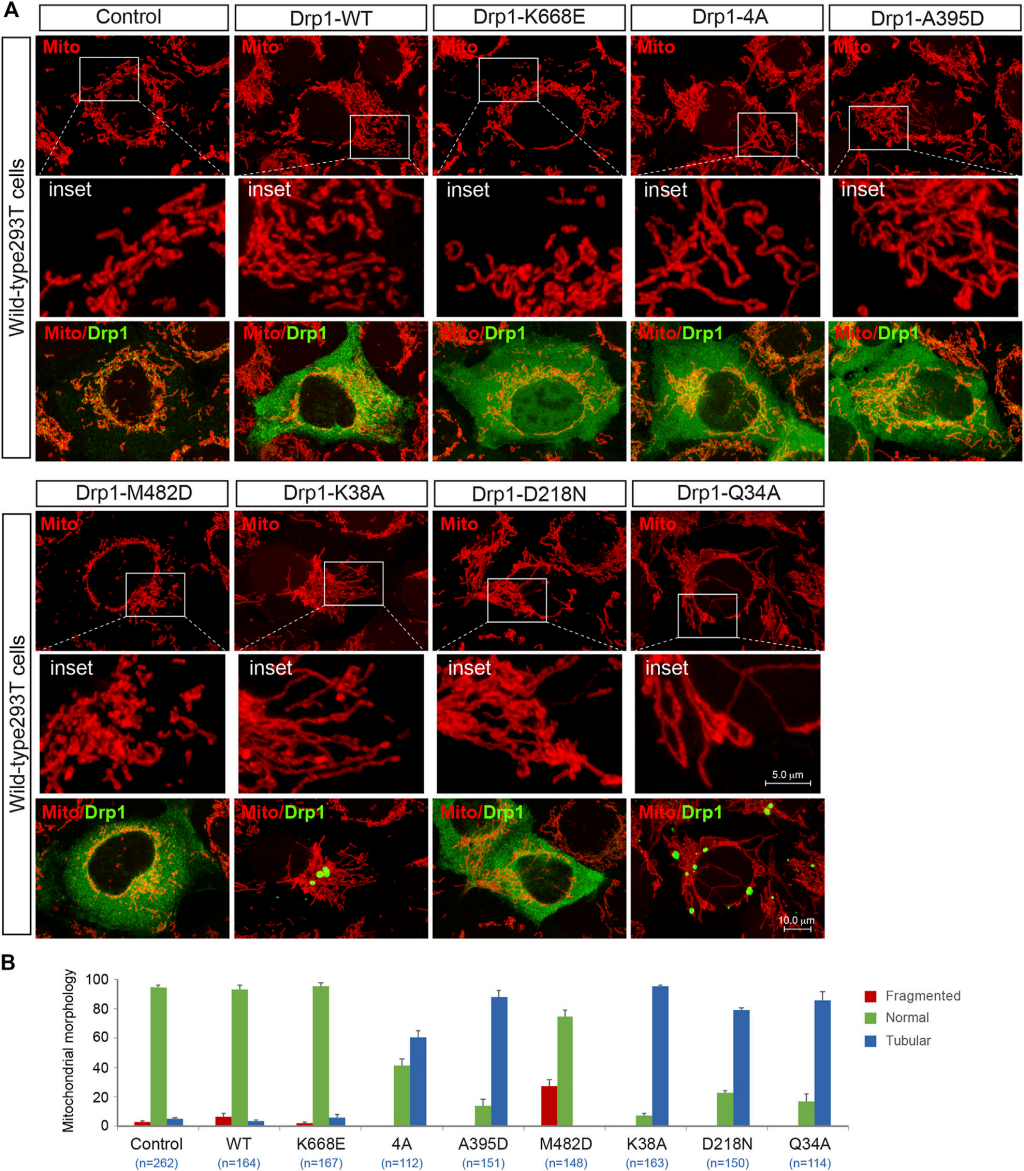
The function-deficient Drp1 mutants impair endogenous Drp1-mediated fission. **(A)** Confocal images of mitochondrial morphology and Drp1 distribution in WT 293T cells transfected with empty vector, exogenous Drp1-WT or Drp1 mutants as indicated. Cells transfected with indicated plasmids were stained with MitoTracker (red) *in vivo* before fixation, followed by immunostaining with anti-Drp1 antibody (green). Insets represent high magnification views of the boxed areas. **(B)** Percentages (mean ± S.E.M.) of cells with indicated mitochondrial morphologies in WT 293T cells transfected with Empty vector, exogenous Drp1-WT or Drp1 mutants shown in **(A)**. n represents the number of cells analyzed.

It is becoming increasingly clear that molecular self-assembly plays a vital role for executing the function of supramolecular complexes. We were therefore interested in exploring whether the fission-incompetent Drp1 mutants affected Drp1-mediated mitochondrial fission via self-assembling with wild-type Drp1. Exogenous expression of Drp1-WT did not affect the morphology of mitochondria in WT 293T cells (Figure 8A, summarized in Figure 8B), consistent with a previous report (Smirnova et al., 1998). With regard to the oligomerization-deficient mutants, expression of the monomeric mutant K668E did not impair endogenous Drp1-mediated fission consistent with its lack of self-assembly property (Frohlich et al., 2013; Michalska et al., 2018), while Drp1-4A and A395D (tetrameric mutants) effectively prevented endogenous Drp1-mediated fission and increased the number of cells with a mitochondrial tubular phenotype (Figure 8A, summarized in Figure 8B), in line with previous studies (Waterham et al., 2007; Frohlich et al., 2013). Conversely, overexpression of the oligomerization-promoting Drp1-M482D mutant stimulated Drp1-mediated fission in WT 293T cells (Figure 8A, summarized in Figure 8B). Finally, expression of the GTPase-deficient Drp1 mutants K38A, D218N, or Q34A (which retain the ability to self-assemble into higher order oligomers) counteracted endogenous Drp1-driven fission, leading to mitochondrial elongation (Figure 8A, summarized in Figure 8B), in agreement with previous studies (Smirnova et al., 1998; Pitts et al., 1999). Collectively, these experiments suggest that all the fission-incompetent Drp1 mutants (except the monomeric mutant K668E) can affect Drp1-driven mitochondrial dynamics via incorporating into the native oligomers to form functionally deficient Drp1 assemblies.

## Discussion

The dynamin-related GTPase Drp1 is an evolutionarily conserved protein and plays a central role in mitochondrial fission. During the process of mitochondrial division, Drp1 is recruited from the cytosol to mitochondria, where it assembles into ring-like structures that consist of oligomeric Drp1 complexes which wrap around and constrict the mitochondrial tubule to drive mitochondrial fission (Yu et al., 2020). The details regarding the dynamic self-assembly state of Drp1 are however still poorly understood and if the minimal self-assembly subunit of Drp1 in intact cells is a dimer (Koirala et al., 2013; Macdonald et al., 2014), tetramer (Shin et al., 1999; Zhu et al., 2004; Bossy et al., 2010; Ugarte-Uribe et al., 2014) or a dynamic dimer-tetramer equilibrium (Chang et al., 2010; Frohlich et al., 2013; Liu and Chan, 2015) remains quite controversial. In this study, we use *in vivo* chemical crosslinking and reveal that the minimal physiological self-assembly subunit of Drp1 is a species of approximately 280 kDa (probably representing the tetrameric form), accompanied by several higher order oligomers, while no monomers or intermediate assemblies (dimers) of Drp1 were observed. This conclusion is in line with previous reports based on *in vitro* chemical crosslinking of cell lysates, blue native PAGE as well as gel filtration analysis (Shin et al., 1999; Zhu et al., 2004; Tanaka et al., 2006; Bossy et al., 2010; Chang et al., 2010; Mishra et al., 2010; Ugarte-Uribe et al., 2014).

Despite extensive efforts, however, the oligomeric state of Drp1 in the cytosol and on mitochondria remains to be clarified. Previous studies indicate that Drp1 in the cytosol exists predominantly as tetramers (Michalska et al., 2018), a mixture of dimers and tetramers (Kwapiszewska et al., 2019) or dimers and higher order oligomers (Clinton et al., 2016). Here, we analyzed the oligomeric state of the cytosol-localized Drp1 via shifting Drp1 from mitochondria to the cytosol by simultaneous knockout of the mitochondrial receptors MIEF1, MIEF2, and Mff. We show that Drp1-WT in the cytosol of these triple receptor knockout cells does not exist as a dimer, but rather as a mixture of multiple self-assembly forms, including tetramers and several higher order oligomers in a dynamic equilibrium, comparable to the oligomeric state seen in intact cells. Conversely, forced recruitment of Drp1 to mitochondria by MIEFs or Mff shifts the equilibrium of the Drp1 oligomeric state towards higher order oligomeric forms, suggesting that receptor-mediated recruitment of the cytosolic Drp1 to the mitochondrial surface stimulates Drp1 higher order oligomerization. In line with this, Mff preferentially binds to higher order oligomeric forms of Drp1, whereas MIEFs bind to a wider-range of Drp1 assembly subunits (including both lower and higher order oligomers) but with an increased association with higher order Drp1 oligomers. Together, these data demonstrate that higher order oligomeric complexes of Drp1 are more prevalent on mitochondria than in the cytosol.

We find this to be exciting and highly challenging data. It should however be kept in mind that many of the experiments presented here rely on crosslinking reagents and we cannot formally exclude that these reagents may in some way affect the Drp1 oligomerization state and its interaction with Mff and MIEFs. Therefore, in future research, it will be important to complement crosslinking experiments with alternative methods to definitely settle this issue. Still, we do not think the *in vivo* crosslinking treatment affected the interactions between Drp1 and its receptors as discussed below. In the supporting co-IP experiments presented in Figures 3D,E, 5B formaldehyde was used as a crosslinking reagent, with consistent results.

Drp1 is a member of the dynamin superfamily of GTPases, but unlike other dynamin family members, it does not contain a specific lipid-binding domain or a transmembrane domain, and therefore the cytosol-localized Drp1 needs to be recruited to the surface of mitochondria through the MOM-anchored receptors in order to execute its fission function. In mammals, Drp1 is recruited to the mitochondrial surface through the MOM-anchored proteins Mff and MIEF1/2 (Gandre-Babbe and van der Bliek, 2008; Otera et al., 2010; Palmer et al., 2011; Zhao et al., 2011; Liu et al., 2013; Loson et al., 2013). Although both Mff and MIEFs can target Drp1 to mitochondria, these two kinds of receptors are functionally different. Mff is believed to recruit active forms of Drp1 to mitochondria, promoting mitochondrial fission (Otera et al., 2010). In contrast, exogenous expression of MIEFs recruits Drp1 to the mitochondrial surface but sequesters it in an inactive form, resulting in mitochondrial elongation (Zhao et al., 2011). However, low to moderate level of MIEFs enhances the interaction between Mff and Drp1, promoting mitochondrial fragmentation (Yu et al., 2017). Together, the emerging data suggest that Drp1-mediated mitochondrial fission can be orchestrated through its selective interaction with Mff and MIEFs (Macdonald et al., 2014; Liu and Chan, 2015; Clinton et al., 2016; Ji et al., 2017), but the molecular details that control the selective binding of Drp1 to Mff and MIEFs remain to be established.

By using a set of genetically modified Drp1 mutants altering the oligomeric state of Drp1 including Drp1-K668E (a monomeric mutant), Drp1-4A and Drp1-A395D (two tetrameric mutants), as well as Drp1-M482D (an oligomerization-promoting mutant), we show that the three oligomerization-defective Drp1 mutants (K668E, 4A, and A395D) fail to bind to and be recruited to mitochondria by Mff. In contrast, Drp1-4A and Drp1-A395D, but not Drp1-K668E, bound to and were recruited by MIEFs to the mitochondrial surface as efficiently as wild-type Drp1. It may be expected that the monomeric mutant K668E does not bind to Mff and MIEFs *in vivo*, because Drp1 does not exist as a monomer or dimer in intact cells (Figures 1A,B). On the other hand, the oligomerization-promoting mutant Drp1-M482D, like wild-type Drp1, robustly interacts with both Mff and MIEFs and distributes both in the cytosol and on mitochondria. Furthermore, the oligomerization-promoting mutant Drp1-M482D is more competent than wild-type Drp1 in mitochondrial fission. Overall, these data suggest that the self-assembly state of Drp1 is crucial for its binding to Mff, while MIEFs are less selective and recruit both oligomerization-deficient and -promoting Drp1 mutants to mitochondria. In support of these observations, *in vivo* crosslinking followed by co-IP revealed that Mff preferentially binds to Drp1 of higher order assembly forms, whereas MIEFs bind to a wider range from lower to higher order oligomers but with an increased association with higher order oligomers. Moreover, GTP binding, hydrolysis and release are also important for Drp1’s selective interaction with Mff or MIEFs. Notably, Drp1-K38A (a GTP-bound mutant lacking GTP hydrolysis and release activity), Drp1-D218N (a GTP-binding defective mutant) and Drp1 Q34A (lacking GTPase activity) failed to interact with and be recruited to mitochondria by Mff, while these GTPase-deficient mutants all robustly interact with and are recruited to the mitochondrial surface by MIEFs as efficiently as wild-type Drp1. These results suggest that MIEFs can recruit Drp1 to mitochondria regardless of its oligomerization state (including lower and higher order forms) or its GTPase activity state. Conversely, Mff selectively recruits higher order oligomeric forms of Drp1 to mitochondria, most likely via a GTPase activity-dependent mechanism, while MIEFs are less selective and recruit both oligomerization-deficient and -promoting Drp1 mutants. MIEFs and Mff coordinately work with distinct functions in Drp1-mediated mitochondrial fission, and this may be one of the reasons why several Drp1 receptors simultaneously exist on the mitochondrial outer membrane.

**FIGURE 9:**
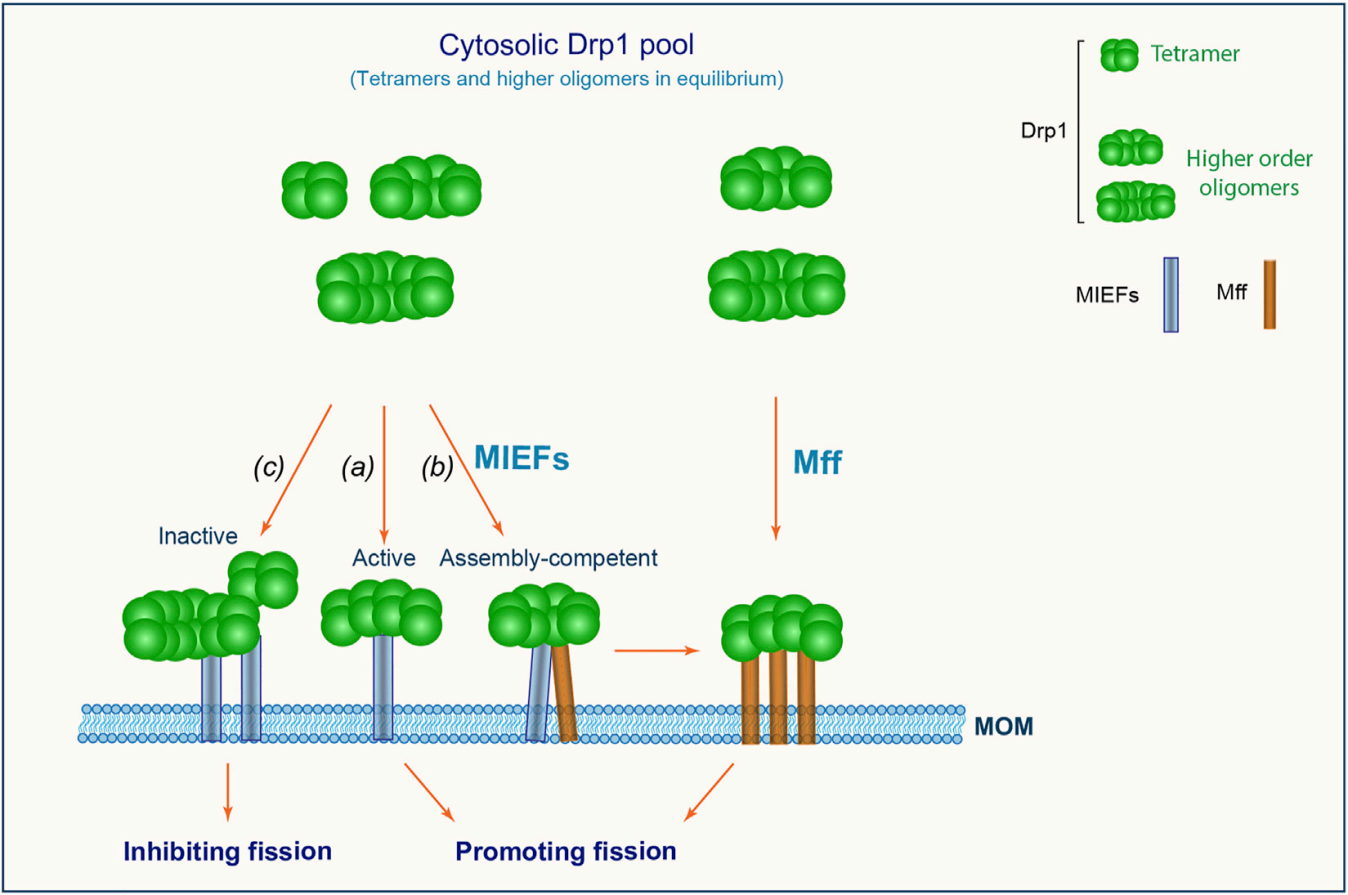
A proposed model for mitochondrial recruitment of Drp1 through MIEFs (MIEF1 and MIEF2) and Mff. Drp1 exists in the cytosol as a reservoir of tetramers and higher order oligomers in active and inactive states. MIEFs can recruit all assembly forms including active and inactive forms of Drp1 to mitochondria. Under physiological conditions, MIEFs bind to active forms of Drp1 facilitating fission **(A)**; MIEFs serve as a platform to facilitate the interaction between Mff and Drp1 **(B)**; However, high levels of MIEFs give rise to Drp1 aggregates and sequester the protein in an inactive state resulting in the inhibition of fission **(C)**. In contrast, Mff alone preferentially recruits GTPase-active and fission-competent forms of Drp1 to mitochondria promoting fission.

Based on the data presented here, we propose a working model (Figure 9), wherein multiple self-assembly forms of Drp1 (including tetramers and higher order oligomers) exist in equilibrium in the cytosol. MIEFs recruit a mixture of Drp1 complexes with lower and higher order assembly forms (including both active and inactive forms) from the cytosol to the mitochondrial surface. At the mitochondrial surface, MIEFs stimulate fission when binding to fission-competent Drp1 and serve as a platform that stimulates oligomerization of assembly-competent Drp1 (for instance tetramers) to produce fission-competent, higher order assemblies of Drp1, and facilitate the GTPase-dependent binding of the fission-competent Drp1 oligomers to Mff, promoting fission. However, when expressed at a high level, MIEFs recruit a large amount of Drp1 to mitochondria and sequester Drp1 in an inactive state as previously reported (Yu et al., 2017), inhibiting fission. In addition, Mff can also selectively and independently recruit a small fraction of GTPase active, higher order oligomers of Drp1 to mitochondria, promoting fission.

## Materials and Methods

### Cell Culture and Reagents

293T (HEK 293T) wild-type and generated knockout 293T cells were cultured at 37°C, and 5% CO_2_ in Dulbecco’s Modified Eagle’s Medium (Sigma-Aldrich) with 10% (vol/vol) fetal bovine serum (FBS, Thermo Fisher Scientific) and 1% Penicillin-Streptomycin antibiotics (Thermo Fisher Scientific).

### Transfection and Expression Constructs

Plasmid transfection was performed using the Lipofectamine™ 2000 transfection reagent (Thermo Fisher Scientific) according to the manufacturer’s protocol. Expression plasmids used in this study were: pcDNA3.1 empty vector, MIEF1-V5 (Zhao et al., 2011), MIEF2-V5 (Liu et al., 2013), Myc-Mff (Gandre-Babbe and van der Bliek, 2008), HA-Drp1-K38A (Smirnova et al., 2001), untagged Drp1-WT and Drp1-Q34A (Yu et al., 2019). Untagged Drp1-K668E, Drp1-4A, Drp1-A395D, Drp1-D218N, and Drp1-M482D were constructed by the QuikChange Lightning Multi Site-Directed Mutagenesis kit (Agilent) using the untagged Drp1-WT as template. All constructs were verified by sequencing experiments. To avoid the side effect of overexpression, cells were transfected with only 0.3–0.5 μg of expression plasmids, and harvested at 16–18 h post-transfection for further analysis.

### Western Blotting and Antibodies

Whole cell lysates from treated cells were dissolved in NuPAGE™ LDS sample buffer (4X) (Thermo Fisher Scientific) and separated by NuPAGE 4–12% Bis-Tris Gel (Thermo Fisher Scientific). Proteins were transferred to polyvinylidene difluoride (PVDF) membranes with the Trans-Blot Turbo system (Bio-Rad). PVDF membranes were blocked with 10% skim milk (Sigma-Aldrich) in TBS, and incubated with indicated primary and secondary antibodies. The target protein bands were detected with the Pierce ECL Western Blotting Substrate (Thermo Fisher Scientific).

In this study, the following primary antibodies were used for immunofluorescence and Western blotting analysis. Mouse monoclonal primary antibodies: V5-tag (Thermo Fisher Scientific); Drp1 and Myc-tag (BD Biosciences) and GAPDH (Santa Cruz). Rabbit polyclonal antibodies: MIEF1 (Zhao et al., 2011), MIEF2, hFis1, and Mff (Atlas Antibodies). Goat polyclonal antibodies: Mff and normal goat IgG (Santa Cruz Biotechnology). The following secondary antibodies were used: The DyLight 488- or 649-conjugated anti-mouse or anti-rabbit IgG antibodies (Vector Laboratories) for immunofluorescence; The peroxidase-conjugated anti-mouse or anti-rabbit IgG antibodies (GE Healthcare) for western blotting analysis.

### Immunofluorescence, Confocal Imaging Acquisition, and Analysis

Cells were seeded on glass coverslips and transfected with indicated plasmids. MitoTracker Red CMXRos (Thermo Fisher Scientific) was used for mitochondria staining *in vivo*. At 16 h post-transfection, cells were incubated with 500 nM MitoTracker for 15 min at 37°C, subsequently fixed with 4% paraformaldehyde (PFA) (Sigma-Aldrich), permeabilized with 0.5% Triton X-100, and blocked in 2% bovine serum albumin (BSA) in PBS for 1 h at room temperature. Thereafter, cells were incubated with indicated primary antibodies overnight at 4°C and DyLight conjugated secondary antibodies for 1 h at room temperature. After three PBS washes, the coverslips were mounted with the Mounting Medium with DAPI (Vector Laboratories). Confocal images were acquired using the SP5 confocal microscopy system (Leica). Quantitative co-localization between Drp1 and mitochondria was analyzed by the Pearson’s correlation coefficient (*PCC*) in the Leica integrated program. To avoid bias, different persons performed the sample preparation, imaging acquisition and data analysis. For 3D surface rendering reconstruction, confocal images were first deconvolved and subsequent 3D surface rendering was performed using the Huygens software (Scientific Volume Imaging B.V.). During image deconvolution, the background estimation and signal-to-noise ratio were set automatically and saved as templates for batch processing images.

### Establishment of Cell Line With Stable Expression of MIEF1-V5

The establishment of cell lines stably expressing MIEF1-V5 was performed as previously described (Yu et al., 2017). 293T cells were transfected with MIEF1-V5 plasmid containing neomycin (G418) resistance. After 24 h post-transfection, the cells were cultured in Dulbecco’s Modified Eagle’s Medium with 10% (vol/vol) fetal bovine serum and G418 (2.5 mg/ml). After 2 weeks selection, the colonies derived from single cells were validated using Western blotting and immunofluorescence.

### Development of Drp1^−/−^ Single-Knockout, Drp1/MIEF1/2^−/−^ Triple-Knockout and MIEF1/2/Mff^−/−^ Triple-Knockout Cell Lines

Drp1^−/−^, Drp1/MIEF1/2^−/−^, and MIEF1/2/Mff ^−/−^ 293T cell lines were established using the CRISPR/Cas9 gene-editing system (Ran et al., 2013) as previously described (Yu et al., 2019; Yu et al., 2017). The CRISPR/Cas9 vector (Addgene plasmid # 48139) including a Cas9 nuclease expression cassette and guide RNA cloning cassette was used in this experiment. The guide RNAs were designed using the online ChopChop software (https://chopchop.rc.fas.harvard.edu/index.php). Drp1 targeting sequence is: GGC​ACA​AAT​AAA​GCA​GGA​CGA​GG; MIEF1 targeting sequence is: GCA​GGC​GCT​GGT​GAG​CGC​AAA​GG; MIEF2 targeting sequence is: GGG​AAG​CGG​CGT​AGC​GAC​GAA​GG; Mff targeting sequence is: GTC​ATC​TGA​CGT​TCC​TTC​AAT​GG. Drp1^−/−^ 293T cells were developed from WT 293T cells with the CRISPR/Cas9 vectors containing the gene-target guide RNA of Drp1 previously described in our published data (Yu et al., 2019). For the generation of MIEF1/2/Mff ^−/−^ triple-knockout cells, 293T cells were transfected with the CRISPR nuclease vectors containing the gene-target guide RNAs (MIEF1, MIEF2 and Mff) for 24 h and selected by puromycin (3 μg/ml) for 48 h. Subsequently, the colonies from single cells were cultured and validated by Western blotting (Figure 1C). The Drp1/MIEF1/2^−/−^ cell line was generated from Drp1^−/−^ 293T cells using the CRISPR/Cas9 vectors containing the gene-target guide RNAs of MIEF1 and MIEF2, and the colonies cultured from single cells were confirmed by Western blotting (Figure 6D).

### *In Vivo* Chemical Crosslinking With DSS and BMH

*In vivo* chemical crosslinking with disuccinimidyl suberate (DSS) (Thermo Fisher Scientific) was performed according to the manufacturer’s protocol and previous description with some modifications (Bofill-Cardona et al., 2000; Zhao et al., 2011). Briefly, cells were transfected with indicated plasmids for 18 h, washed three times in phosphate buffered saline (PBS) with Ca^2+^/Mg^2+^, and incubated with freshly-prepared 1 mM DSS or DMSO (as control) in PBS with Ca^2+^/Mg^2+^ for 3 h at room temperature. The reaction was quenched by 50 mM Tris (pH 7.5) for 15 min at room temperature. The samples were dissolved in NuPAGE™ LDS sample buffer (4X) (Invitrogen) and analyzed by Western blotting.

*In vivo* chemical crosslinking with bismaleimidohexane (BMH) (Thermo Fisher Scientific) was carried out according to the manufacturer’s procedure. BMH powder was dissolved firstly in DMSO and mixed with PBS to a 50 µM final concentration, and incubated with cultured cells at room temperature. This reaction was quenched by 50 mM dithiothreitol (DTT) at different time points. After removing the reaction solution, treated cells were added to NuPAGE™ LDS sample buffer (4X) (Invitrogen) and subjected to further Western blotting analysis.

### Co-Immunoprecipitation (Co-IP)

Co-IP experiments were performed as previously described (Liu et al., 2013; Yu et al., 2017). *In vivo* crosslinking with 1% formaldehyde (FA) was performed for 10 min at room temperature, and quenched by 100 mM glycine. For *in vivo* crosslinking with DSS followed by co-IP, living cells were treated with DSS (1 mM for 3 h) and quenched by 50 mM Tris (pH 7.5) for 15 min at room temperature. Thereafter cell lysates suspended in lysis buffer (1% NP-40 with protease inhibitor cocktail complete EDTA-free (Roche Diagnostics)) were subjected to co-IP experiments. For co-IP of V5-tagged MIEF1 and MIEF2 or Myc-tagged Mff, anti-V5 or anti-Myc agarose beads (Novus Biologicals) were incubated with the cell lysates for 2 h at room temperature. For co-IP of endogenous Mff, Dynabeads™ protein G beads (Thermo Fisher Scientific) pre-incubated with 2 μg goat anti-Mff antibody were added to cell lysates overnight at 4°C. Following three washes with lysis buffer for the beads, the bead-binding proteins were dissolved in sample buffer and subsequently subjected to Western blotting analysis.

### Statistical Analysis

Data are collected from at least three independent experiments for statistical analysis, and presented as mean ± S.E.M. (Standard Error of the Mean). Statistical analysis between two groups was carried out using the Student’s t-test. A *p*-value equal to or less than 0.05 was considered to be statistically significant.

## Data Availability

The original contributions presented in the study are included in the article/Supplementary Material. Further inquiries can be directed to the corresponding authors.
