# Supplementary material for: The Molecular Assembly State of Drp1 Controls its Association With the Mitochondrial Recruitment Receptors Mff and MIEF1/2
Source: Front Cell Dev Biol. 2021 Nov 5;9:706687. doi: 10.3389/fcell.2021.706687 (PMC8602864; doi:10.3389/fcell.2021.706687)
Supplement: Supplementary file 1 [file DataSheet1.docx]

**Supplemental information**

**The** **molecular assembly state of Drp1** **controls its association with the mitochondrial recruitment receptors Mff and MIEF1/2**

Rong Yu^1^, Shao-Bo Jin^2^, Maria Ankarcrona^3^, **Urban Lendahl**^2,3^**,** Monica Nistér^1*†^ and Jian Zhao^1*†^

^1^ Department of Oncology-Pathology, Karolinska Institutet, BioClinicum, Visionsgatan 4,

## Karolinska University Hospital Solna, SE-171 64 Solna, Sweden;

## ^2^ Department of Cell and Molecular Biology, Karolinska Institutet, Biomedicum, Solnavägen 9, SE-171 77 Stockholm, Sweden.

^3^ Department of Neurobiology, Care Sciences and Society, Center for Alzheimer Research, Division of Neurogeriatrics, Karolinska Institutet, BioClinicum J9:20, Visionsgatan 4, SE-171 64 Solna, Sweden;

## ^*^Correspondence: [monica.nister@ki.se](mailto:monica.nister@ki.se); [jian.zhao@ki.se](mailto:jian.zhao@ki.se)

^†^These authors contributed equally to this work as senior authors

**Supporting information**

**
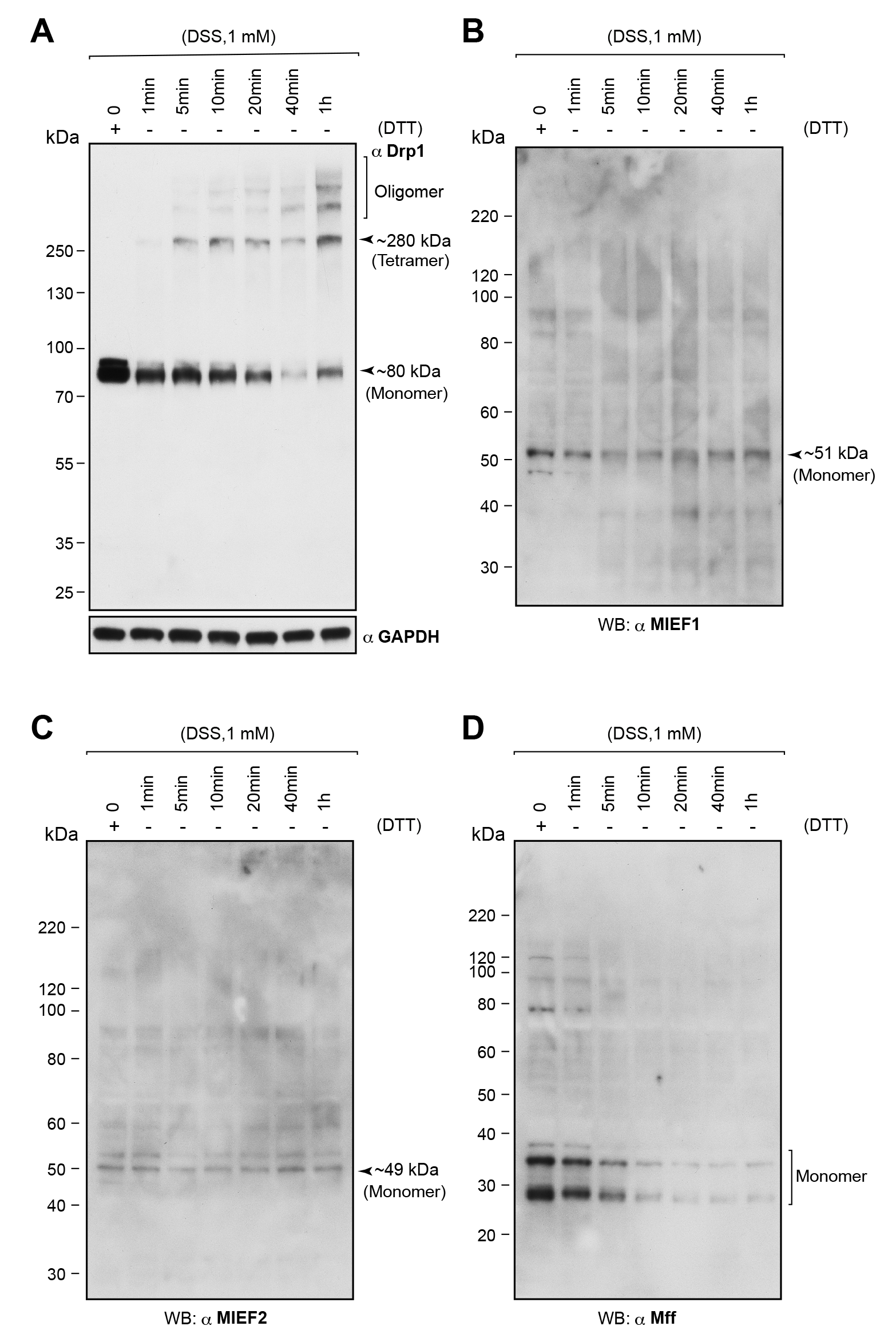
**

**Figure S1. The monomeric form of Drp1 gradually disappeared in a time-dependent manner, and multiple higher assembly units of Drp1 were observed as bands ranging from a minimal self-assembly subunit at ~280 kDa to several higher order oligomers upon treatment with DSS.** 293T cells were treated with the cell-permeable crosslinking reagent DSS (1 mM) for a time course as indicated, and cell lysates were analyzed by Western blotting using indicated conditions and antibodies. GAPDH was used as loading control in the Western blots.

**
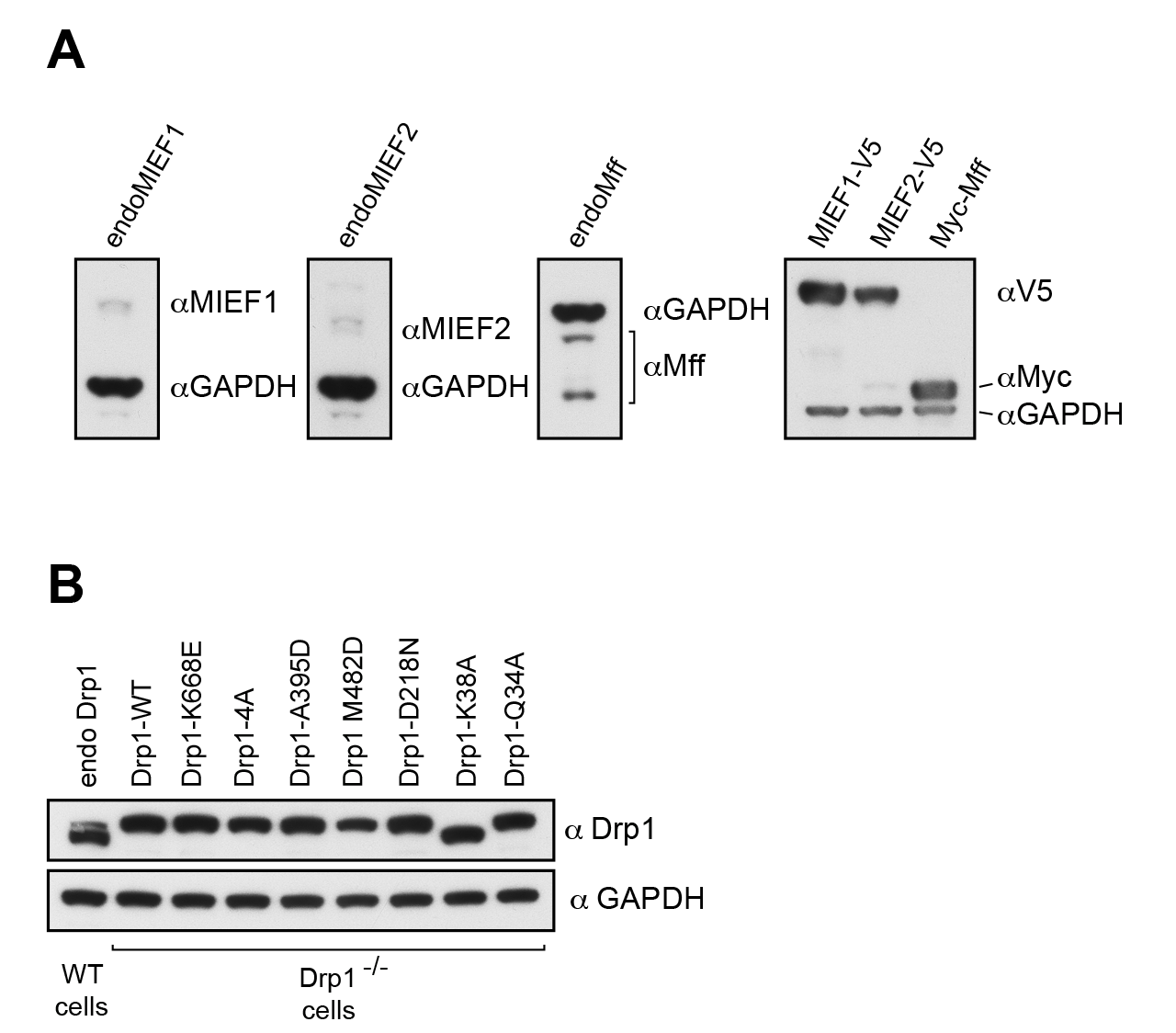
**

**Figure S2. Expression levels of Drp1 WT and mutants, MIEF1-V5, MIEF2-V5 and Myc-Mff.** (**A**) 293T cells were transfected with 0.5 µg of empty vector, MIEF1-V5, MIEF2-V5 or Myc-Mff for 18 h, followed by Western blotting with indicated antibodies. (**B**) WT or Drp1^−/−^ 293T cells were transfected with 0.5 µg of empty vector, Drp1 WT or indicated Drp1 mutants for 18 h, followed by Western blotting with indicated antibodies.


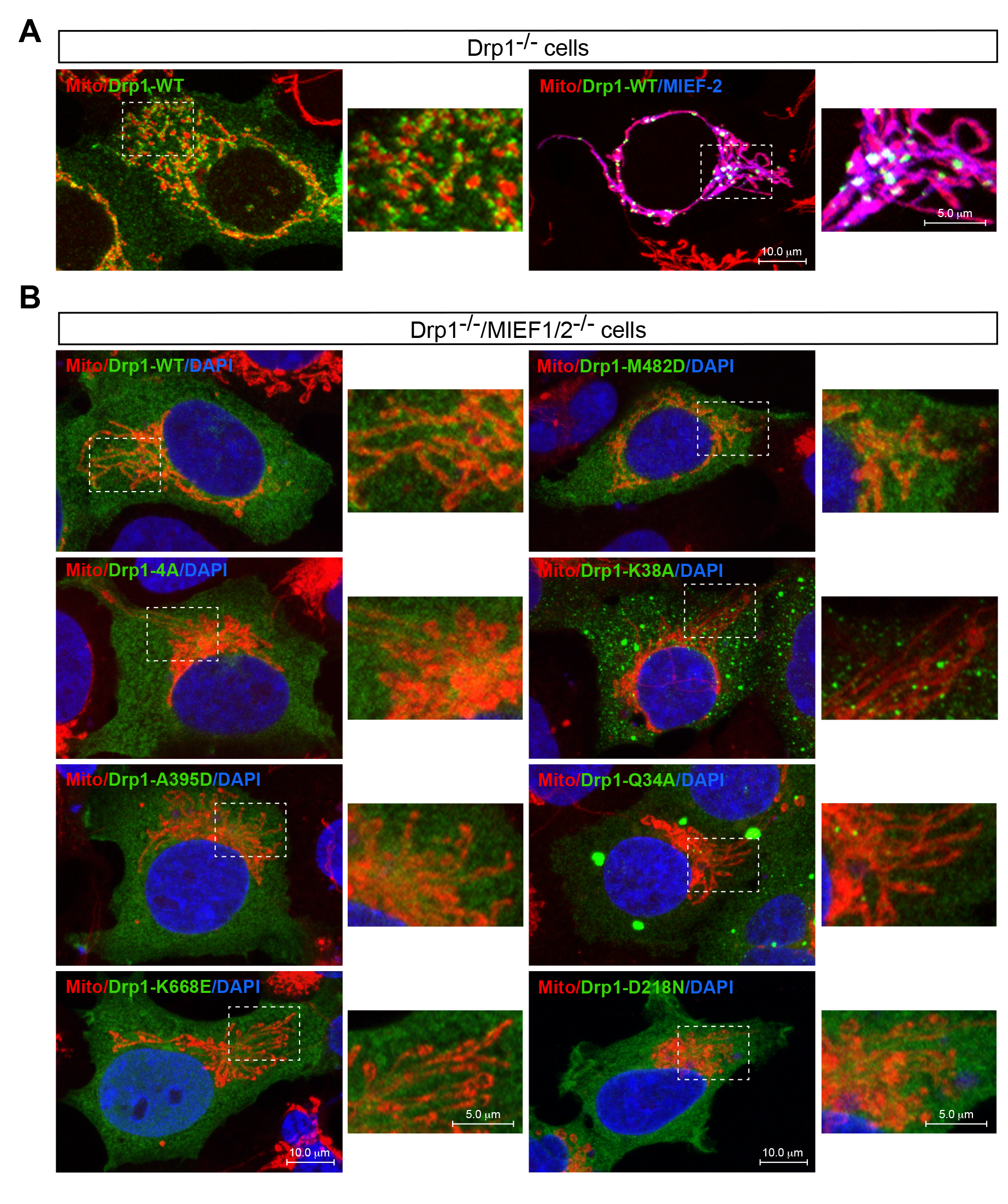


**Figure S3****.** **Drp1 oligomerization-deficient or GTPase-deficient mutants were not recruited to mitochondria in the absence of endogenous MIEF1 and MIEF2.** (**A**) Confocal images of mitochondrial morphology and Drp1 distribution in **Drp1**^−/−^ 293T cells transfected with exogenous Drp1-WT or co-transfected with Drp1-WT and MIEF2-V5. Cells transfected with indicated plasmids were stained with MitoTracker (red) in vivo before fixation, followed by immunostaining with anti-Drp1 antibody (green) and V5 antibody (blue). Insets represent high magnification views of the boxed areas. (Yellow color indicates co-localization of Drp1 and mitochondria. White color indicates co-localization of mitochondria, Drp1 and endogenous MIEF-2 protein.) (**B**) Confocal images of mitochondrial morphology and Drp1 distribution in **Drp1**^−/−^**/MIEF1/2**^−/−^ 293T cells transfected with exogenous Drp1-WT or Drp1 mutants as indicated. Cells transfected with indicated plasmids were stained with MitoTracker (red) *in vivo* before fixation, followed by immunostaining with anti-Drp1 antibody (green). Nuclei are stained with DAPI (blue) Insets represent high magnification views of the boxed areas. The GTPase-defective Drp1 mutants (K38A, Q34A) show no co-localization with mitochondria. Mitochondrial localization of the monomeric mutant (K668E) is decreased.
